# Maintaining the Outcomes of a Successful Weight Gain Prevention Intervention in Mid-Age Women: Two Year Results from the 40-Something Randomized Control Trial

**DOI:** 10.3390/nu11051100

**Published:** 2019-05-17

**Authors:** Lauren T. Williams, Clare E. Collins, Philip J. Morgan, Jenna L. Hollis

**Affiliations:** 1 Menzies Health Institute Queensland, Griffith University, Southport, QLD 4222, Australia; 2 School of Health Sciences, Faculty of Health and Medicine, University of Newcastle, Callaghan, NSW 2308, Australia; clare.collins@newcastle.edu.au (C.E.C.); Jenna.Hollis@health.nsw.gov.au (J.L.H.); 3 Priority Research Centre in Physical Activity and Nutrition, University of Newcastle, Callaghan, NSW 2308, Australia; philip.morgan@newcastle.edu.au; 4 School of Education, Faculty of Education and Arts, University of Newcastle, Callaghan, NSW 2308, Australia

**Keywords:** ageing, menopause, bodyweight changes, obesity, dietitian, quality of life, behavior modification, motivational interviewing, social cognitive theory, mid-age women, waist circumference

## Abstract

Despite the life stage of menopause being identified as a high risk for weight gain, there are few obesity prevention interventions for this target group, and no evidence on maintenance of intervention effects after intervention support is withdrawn. In the 40-Something Randomized Controlled Trial (RCT) (ACTRN12611000064909), a five-consultation health professional (dietitian and exercise physiologist) obesity prevention intervention, using motivational interviewing principles (MI) over 12 months, achieved significantly greater weight loss than a self-directed intervention (SDI) (tailored written material) in 54 non-obese (body mass index (BMI): 18.5–29.9 kg/m^2^), premenopausal women (44–50 years). The aim of the current paper is to report on whether the intervention effects were maintained at two years. Anthropometric, biochemical and health behavior data were collected at baseline, 12 months (end of intervention) and 24 months (end of maintenance period). Forty participants (22 = MI, 18 = SDI) who completed all measures to 12 months were invited to participate in the monitoring phase and 30 (MI = 16, SDI = 14) consented. The primary outcome of weight at 24 months was assessed using intention to treat principles (*n* = 54), adjusting for baseline weight. The MI group had a significantly lower weight at 24 months (64.6 kg, 95% CI: 63.2, 66.6, *p* = 0.015) compared with the SDI group (67.3 kg, 95% CI: 65.7, 68.8), and the secondary outcomes of percentage body fat and waist circumference were also significantly lower in the MI group. The low-intensity, health professional weight control intervention utilizing MI principles was more efficacious in maintaining a significant weight loss compared to a self-directed intervention, and both were successful in preventing obesity.

## 1. Introduction 

The primacy of obesity as a major health problem is well established and effective interventions are urgently needed. Obesity prevention interventions at life stages associated with high risk of weight gain, such as menopause, are important in decreasing obesity incidence. Menopause can be viewed as a physiological ageing process, instigated by loss of ovarian function and concomitant hormonal changes, particularly estrogen insufficiency [1]. One consequence of decreased circulating estrogen levels is the increased susceptibility to the deposition and redistribution of fat abdominally [2]. Because this occurs independent of body mass index (BMI) category, it is possible for women within a healthy BMI range to develop abdominal obesity [3]. Preventing weight gain at menopause has the potential to minimize metabolic syndrome and associated co-morbidities, such as cardiovascular disease, that increase in prevalence in post-menopausal women [4]. 

Despite the priority for weight gain prevention at this life stage, few obesity prevention interventions have been designed for menopausal women. A systematic review of lifestyle interventions targeting weight changes in the menopause transition published between 2003 and 2011 found only three studies [5], two of which conducted an exercise-only intervention [6,7]. The remaining study, the Women’s Healthy Lifestyle Project (WHLP), demonstrated that a lifestyle intervention aimed at educating participants about eating and exercise delivered over a 54 month period was successful in preventing increases in weight, triglycerides and glucose associated with menopause and achieved a decreased waist circumference [8]. No weight gain prevention interventions for menopausal women have included a passive maintenance period. A period of monitoring post-intervention allows the assessment of whether the dietary intake and physical activity behavior change achieved by the intervention has been sustained as part of the participants’ lifestyle [9]. There is, therefore, a need for research that examines the long-term success of interventions and the characteristics of that success [10].

The 40-Something Randomized Controlled Trial (RCT) tested a relatively low-intensity health professional intervention aimed at preventing weight gain in 45–50 year-old women who were on the brink of the menopause transition and not obese [11]. The intervention aimed to intrinsically motivate the women to permanently change their diet and physical activity behaviors to achieve weight control. Motivational interviewing (MI) was used as a counselling strategy, given evidence of its effectiveness in weight control [12] and in improving diet and physical activity [13]. At the end of the 12 month intervention, women who had received the MI based intervention had a significantly lower mean (SD) body weight (MI: 65.6 (8.5) kg versus SDI: 67.4 (6.7) kg, 95% CI: −3.490; −0.145, *p* = 0.034) and diastolic blood pressure (MI: 74.4 (5.5) mmHg versus 76.8 (8.7) mmHg, 95% CI: −5.572; −0.175, *p* = 0.037), after adjusting for baseline measures, than women who received a self-directed intervention (SDI) with tailored print materials only [14]. The diets of the MI group were more nutrient dense with respect to food sources of iron and potassium, and they consumed more fruit [15]. However, such improvements in behaviors and health outcomes are only important if the results can be sustained over time, given the high proportion of weight management intervention studies that show weight rebound [16,17]. Measurement of effect maintenance for at least 12 months is needed to evaluate the long-term effect of interventions in improving chronic disease health trajectories [18].

The aim of the current study was to examine the 24 month primary (weight) and secondary (body composition, blood pressure and quality of life) outcomes of the 40-Something study 12 months after withdrawal of intervention support. Given that intrinsic motivation has been shown to support sustained changes in weight control behaviors in mid-aged women [19] we hypothesized that the use of MI in the health professional consultations would assist behavior change maintenance. It was hypothesized that there would be a statistically significant (*p* < 0.05) difference of at least 3.5 kg between the groups at 24 months. This figure was based on two assumptions. Firstly, that the women in the MI intervention group would achieve the recommended intervention goals, which were a net weight change of 0 for healthy weight women and a net loss of 5 kg for overweight women (the average amount overweight women needed to lose in order to achieve a BMI in the healthy weight range). Given the sample was stratified to achieve equal numbers of overweight and healthy weight women, half the MI group should lose 5 kg and the other half should lose 0, resulting in a net weight loss of that group of 2.5 kg. The second assumption was that the SDI (control) group would gain one kg in weight (based on observational data from a population-based study of women of the same age [11].

## 2. Materials and Methods

### 2.1. Study Design and Participants in the 40-Something Intervention Phase

A parallel-group RCT design was used to compare the 12 month health professional-led intervention using Motivational Interviewing with a tailored written information self-directed intervention. The study received ethical approval from the University of [blinded for peer review] Human Research Ethics Committee (H2010-0030) and was registered with the Australian New Zealand Clinical Trials Registry (reference number ACTRN12611000064909). The authors have previously published the detailed methods of the study [11] and the weight, diet and physical activity results of the 12 month, 40-Something weight gain prevention intervention [14,15]. 

**Figure 1:**
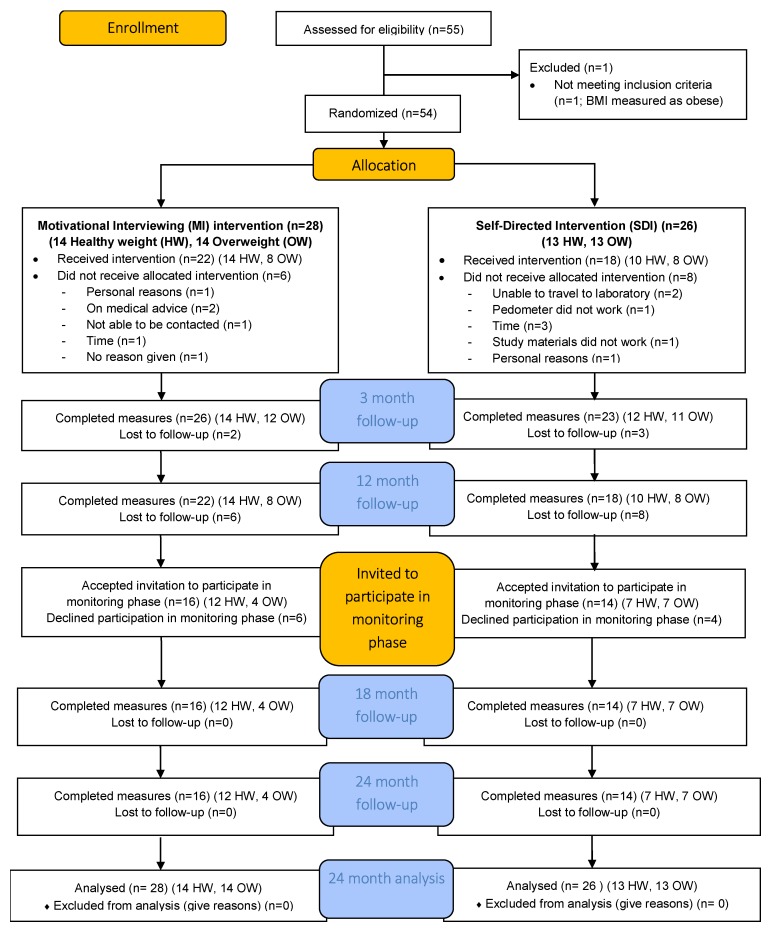
CONSORT flow diagram for the 40-Something study.

In brief, 54 women were recruited to the 12 month intervention study from the Newcastle region, Australia. Recruitment channels included radio and television interviews, newspaper articles and flyers placed in medical clinics and University notice boards. Those women expressing interest were screened against inclusion criteria (women aged 44–50 years at baseline, with a BMI between 18.5–29.99 kg/m^2^, menstruated within the preceding three months, and free of major diseases including cardiovascular disease, diabetes and cancer) by telephone interview between May and July of 2010. Women were categorized by BMI as healthy weight (18.5–24.99 kg/m^2^) or overweight (25.0–29.99 kg/m^2^), according to their baseline measures, then randomly assigned to the MI or SDI group using computer-generated random number sequences (one list for the healthy weight BMI range and one for the overweight BMI range to stratify the sample). The study was powered for a between group difference of 3.5 kg, assuming equal sample sizes, 80% power, a significance level of 0.05 and 20% attrition over the 24 months. Measures for the intervention phase were collected at baseline, 3 and 12 months, and at 18 and 24 months in the monitoring phase [11]. The CONsolidated Standards of Reporting Trials (CONSORT) diagram showing participation through the 24 month study is shown in Figure 1. 

Trial registration: Australian New Zealand Clinical Trials Registry (reference number ACTRN12611000064909).

### 2.2. The 40 Something Intervention Description

The interventions were framed according to Social Cognitive Theory (SCT) principles [20], which was used to understand why and how people change individual health behaviors within their social and physical environments, and to design an intervention capable of intrinsically motivating the participants to make diet and physical activity behavioral changes required for weight control and to sustain those changes over time. All participants, regardless of allocation, were provided with achievable weight goals according to their baseline BMI. Women in the healthy weight range (18.5–24.99 kg/m^2^) at baseline were advised to maintain their weight within 1 kg for the duration of the study by consuming 8300 kJ/day, performing 150 min of physical activity/week and walking 10,000 steps/day. Women in the overweight BMI range (25–29.99 kg/m^2^) were given a weight loss target that would allow then to achieve a BMI in the healthy weight range by consuming 6300 kJ/per day, performing 250 min of physical activity/week and walking 10,000 steps/day [11]. Once healthy weight was achieved, they were then provided with the goals and advice given to the healthy weight women. Both MI and SDI interventions incorporated the SCT components of behavioral capability (knowledge and skills required to perform a behavior) by providing written (SDI and MI groups) and verbal (MI only) with the information to meet these goals, with tips for achieving weight control, healthy eating and physical activity. Self-control was promoted by encouraging all women to regulate and self-monitor their behaviors through self-monitoring tools and materials to assess attainment of achievable goals for weight, dietary intake and physical activity. Written materials were given to all participants at the baseline measurement session prior to group allocation.

Participants in both the MI and SDI groups were provided with documented behavior change strategies individually tailored to the results of their baseline and three month dietary intake and physical activity measures. For the MI group, these strategies were developed and revised by the participant during 4 × 60 min face-to-face consultations with a Dietitian and a 1 × 60 min consultation with an Exercise Physiologist, using a guiding method of communication to create an environment where the client felt comfortable to explore and resolve ambivalence to behavior change [21]. The health professionals delivering the MI intervention were registered Medicare providers who reported undertaking additional training in MI (including online courses, self-directed reading and workshops) [14]. The health professionals used consultation protocols that included activities and discussions designed to build self-efficacy (a belief that a person has control over, and can successfully perform, a behavior [20]) by exploring and drawing on the participant’s strengths and past successes and understanding outcome expectations (to determine the consequence of a behavior) through open-ended questions and a decisional balance activity to identify both the advantages and disadvantages of making a behavior change. The SDI group were mailed a document containing their weight, diet and physical activity goals, along with suggested behavior change strategies, tailored to their measures at baseline and 3 and 12 months by the same health professionals.

### 2.3. Study Design and Participants in the 40-Something Monitoring Phase

The monitoring phase commenced at the end of the 12 month intervention. The 40 women who completed all components of the intervention phase, including all 12 month measures, were invited to participate and to provide informed, written consent. All women were treated identically in the monitoring phase, regardless of original group allocation. The monitoring study commenced with the provision of individualized 12 month intervention results and tailored weight, diet and physical activity goals were provided in writing to all participants. While participants received no further appointments or new materials in this phase, they were encouraged to continue self-monitoring their weight and diet and physical activity. After the visit to the laboratory to collect the 12 month measures, contact with participants was limited to the measurement sessions at 18 and 24 months and being mailed one study newsletter to maintain a sense of study involvement. Researchers remained blinded to the allocation condition (except for JH, who had delivered the Dietitian component of the MI intervention). The women were reminded not to reveal their original allocation group when they attended the measures.

### 2.4. Outcome Measures

Outcome measures were collected by trained assistants at baseline, 12, 18 and 24 months. Participants were measured in light indoor clothing after an overnight fast and voiding of urine using a bioelectrical impedance analyzer Omron HBR-500 Body Composition Monitor with Scales (Omron Healthcare Co. Ltd., Kyoto Japan) for the primary outcome measure of weight and the secondary outcome measures of fat mass and lean mass [11]. The secondary outcome measure of waist circumference was collected with a non-extensible steel tape to measure at the level of the mid-point between the lower costal border and the iliac crest (KDS F10-02, KDS Corporation, Osaka, Japan) [11]. Systolic blood pressure (SBP) and diastolic blood pressure (DBP) and resting heart rate were measured after participants rested for at least five minutes using a NISSEI/DS-105E digital electronic blood pressure monitor (Nihon Seimitsu Sokki Co. Ltd., Gunma, Japan) under standardized procedures [11]. Visceral adipose tissue (VAT) area was collected at 12 months (after the purchase of an InBody720 Body Composition Analyzer (InBody, Seoul, Korea)) 18 and 24 months.

Menopause symptoms, health behaviors and attitudes (including physical activity measured by the International Physical Activity Questionnaire (IPAQ) short version [22]) and health-related quality of life measures (Short Form-36) were collected, along with demographic data using a written survey at baseline, 12 and 24 months. The SF-36 is an eight-scale health profile used to generate a physical component summary (PCS) and a mental component summary (MCS) score [23]. These scores were compared to published age and gender specific population norms for mid-age women aged 45–49 years, allowing for a more meaningful comparison standardized to the Australian population [24]. Participants were provided with a purpose-developed calendar to record each day of menstrual bleeding for the 24 months of the study. Women with no menstrual bleeding for ≥3 and <12 months were categorized as peri-menopausal; women with no menstrual periods for ≥12 months were categorized as post-menopausal [25].

### 2.5. Statistical Analyses

One-way analysis of variance and Chi-square tests on continuous and categorical variables, respectively, were used to compare differences in characteristics of those who participated in the monitoring phase versus non-participants. Continuous variables were checked for the plausibility of outliers, then for the normality of distribution using the Shapiro-Wilk test. Parametric and non-parametric tests were applied to normally and non-normally distributed variables respectively. Intention to treat (ITT) principles were applied to statistical analyses of the outcome variables on the 54 women invited to participate in the original trial, using the last observation carried forward method to impute values for missing data. All data analyses were performed using SPSS version 22 (IBM Corp., Armonk, NY, USA). Statistical significance was set at 0.05.

As in the case of the 12 month results, between-group differences for primary and secondary outcomes were tested using analysis of covariance, with baseline values as covariates, and intervention group as the fixed factor. VAT was the exception with 12 month results used as a covariate given this was the first data collection point. Weight goal attainment was calculated for each participant and scored as a dichotomous variable (achieved/not achieved). Women who had a healthy weight BMI at baseline attained their goal if they gained less than one kilogram from baseline. Women who had an overweight BMI at baseline attained their weight goal if their 24 month weight was within the healthy weight range. The proportion of those achieving their goal in each intervention group was compared using Chi-square tests.

## 3. Results

### 3.1. Participants

Forty women (22 MI, 18 SDI) completed the intervention phase. Of these, 30 consented and completed the full monitoring phase, with approximately equal participation by those previously allocated to the MI group (*n* = 16) and the SDI group (*n* = 14). Table 1 compares the baseline participant characteristics for the 30 women who participated to 24 months, the ten women who completed the intervention to 12 months but did not participate in monitoring and the 14 women who commenced the study but did not complete the intervention phase (five withdrew before the 3 month measures and the other nine withdrew between the 3 month and 12 month measures). There were significant differences at baseline between these three groups for two variables only: BMI category (but not BMI as a continuous variable), with more of the overweight (BMI 25–29.99 kg/m^2^) women withdrawing before 12 months, and for ability to manage based on income, with more of the women who found it difficult to impossible to manage on their income withdrawing before 12 months.

All women were premenopausal and not taking hormone replacement therapy at baseline according to the recruitment criteria. Of the 30 women who completed the monitoring study, 19 remained pre-menopausal at 24 months, two were peri-menopausal, three were post-menopausal and the remaining six were of unknown menopausal status because they started using a device that affected their periods during the study (data not shown). It was not possible to analyze for menopause symptoms given the infrequent responses due to the high proportion of women remaining pre-menopausal or commencing device use.

By 24 months, three of the women who had been in the overweight category at baseline had a BMI in the healthy weight range, giving 22 women in the healthy weight and eight in the overweight categories at 24 months. Only six of the 30 women who completed the study to two years (2 MI and 4 SDI) did not attain their weight goals (being within 1 kg of their baseline weight or losing sufficient weight to move to the healthy weight range for women who were healthy weight or overweight at baseline, respectively). In this completers analysis, there was no significant difference between the MI and SDI groups in goal attainment. No participants became obese during the 2 year study period.

### 3.2. ITT Weight Analyses

The significant intervention effect previously reported for weight change in the 12 month intervention phase [14] was maintained at 24 months, with the MI group having a significantly lower weight than the SDI group (*p* = 0.024) (Table 2). However, the hypothesized difference of 3.5 kg (based on MI women losing 2.5 kg and maintaining that loss, and SDI women gaining 0.5 kg per year) was not achieved. While the MI women lost close to the predicted amount of 2.5 kg, and maintained that loss, the SDI women did not gain weight at the predicted rate of 0.5 kg per year; rather, they maintained their baseline weight. When the groups were analysed according to baseline BMI category, no significant intervention effects were observed for the women who were overweight at baseline, only for the women who were a healthy weight at baseline and were thus responsible for the overall group effect as shown in Table 2.

**Figure 2:**
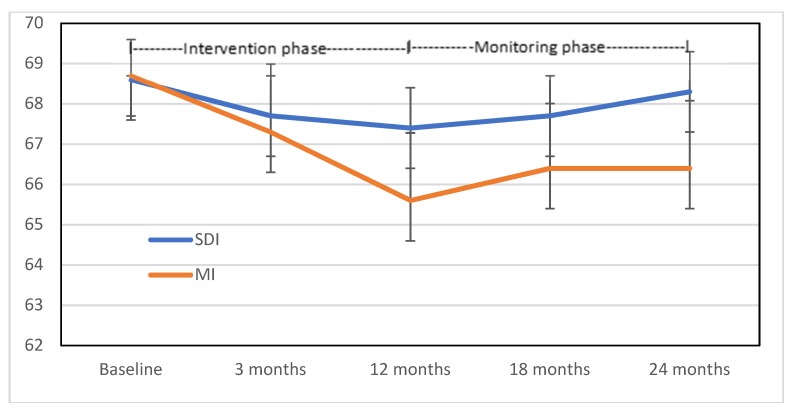
Mean (95% CI) weight (kg) for the Motivational Interviewing (MI) group and the Self-Directed Intervention (SDI) group over 24 months (ITT analysis) (*n* = 54).

A graph showing the pattern of weight change of the 54 participants across phase one (intervention) and phase two (monitoring only) is shown in Figure 2. The women in the SDI group lost weight by the end of the intervention phase but regained virtually all of it by 24 months. The women in the MI group, who had lost significantly more weight than the SDI women by the end of the intervention phase, had regained some of that weight by 18 months, but then stabilized and maintained that weight to 24 months.

### 3.3. Secondary Outcome Measures: Physical Measures

In line with the significant weight changes, percentage of body fat and waist circumference also showed a significant intervention effect at 24 months in adjusted analyses (Table 3). Waist circumference had also been significant at 12 months [14]. The SDI women decreased their waist measurement by 0.7 cm over 24 months, while the MI women decreased by 4.3 cm. There were no significant differences for percentage of lean muscle mass, or for VAT, between the two intervention groups. While an intervention effect for mean DBP was observed at 12 months [14], this result was no longer significant at 24 months.

### 3.4. Secondary Outcome Measures: Quality of Life

The summary variables for physical (PCS) and mental (MCS) health from the SF-36 quality of life measure (see Table 4), were non-normally distributed. Results from the Mann Whitney *U* tests for independent samples showed there to be no difference between intervention groups in the scores at any of the timepoints, nor was there a significant change across time in the Mann Whitney *U* tests on related samples.

## 4. Discussion

The 40-Something RCT showed that a relatively low-intensity health professional intervention, using a motivational interviewing counselling style, successfully prevented weight gain to two years in a group of women at significant risk of weight gain due to the ageing life stage of menopause. There was also evidence that waist circumference, one of the risk factors for metabolic syndrome, was positively influenced by the intervention. One year after intervention cessation, the women who had received the MI intervention had regained only a fraction of a kilogram and remained stable at their new weight for at least six months. The SDI group, which acted as a control, regained the weight lost; however, they only did so to their baseline weight and, therefore, successfully prevented weight gain and the development of obesity. When the results were analyzed according to baseline BMI, there were differences according to intervention group. The healthy weight MI women lost 1.9 kg over the 24 months while the healthy weight SDI women gained 0.1 kg with a p value of 0.031. The women who were overweight at baseline lost weight regardless of allocation group (2.5 kg in the MI group versus 0.8 kg in the SDI group), a between-intervention group difference that was not statistically significant. Thus, the group intervention effects were due to weight loss by the women who were in the healthy weight BMI category at baseline. This observation was also evident at the end of the intervention phase, and we previously conjectured that weight loss may have been more of a challenge for the overweight women due to higher abdominal adiposity, and a combination of the lifestyle and environmental factors that led to them being affected by overweight at baseline [14]. These factors may similarly make weight loss maintenance more challenging once people have had a prior experience of being overweight. In contrast, the healthy weight women who were advised only to maintain weight, lost weight, perhaps because it was metabolically easier for them to do so. Despite losing weight, they remained within the healthy weight range rather than becoming underweight.

Data from a population-based longitudinal study, the Australian Longitudinal Study on Women’s Health (ALSWH), shows that the typical pattern for this demographic group is one of gradual weight gain [26]. The ALSWH found that women aged 45–50 years at baseline gained 0.5 kg/year and 2.1% of the cohort who were overweight at baseline became obese over a two year period [26]. This pattern occurred despite 74% of the cohort reporting to be actively attempting to control their weight [27]. Had the women in the current study not participated in either study arm of the RCT they are likely to have followed the population pattern of weight gain. Instead, every participant who completed the study to 24 months, both intervention and control, avoided becoming obese. Eighty percent of these women achieved their weight goals (to maintain or lose weight as specified according to their baseline BMI), with several moving from an overweight BMI at baseline to a healthy weight BMI at 24 months, and this did not vary by intervention type. It should be noted that this is a completers analysis, given that it was not possible to apply ITT principles to the weight goal data.

In line with the weight changes, significant intervention effects were observed for waist circumference and percentage of body fat, but not for percentage of lean mass or for VAT. While the VAT figures showed a trend toward a decrease in the intervention group, the high variability observed meant that the result was not statistically significant. It is important to note that the study sample size was not powered for this variable. Given that the pattern of body composition change at menopause is characterized by abdominal fat deposition and loss of lean body mass [2], the 40-Something results are metabolically important, particularly for the MI intervention group. While both groups maintained lean muscle mass, the MI group also decreased their body fat percentage and waist circumference, which is the ideal body composition profile for weight gain prevention at this life stage. Interventions for menopausal women should specifically address body composition, considering that weight change is only a proxy measure of fat gain and does not assess the abdominal fat redistribution characteristic of estrogen deficiency.

While there was a trend toward decreased DBP in the MI group, this did not reach statistical significance at 24 months, despite having been significant at 12 months. Similarly, there was no difference in the intervention groups in the summary scores on the SF 36 nor was there any difference across time points. However, this result needs to be considered in the context of SF 36 results for the entire population. At 24 months, the women in the MI group had a PCS of 51.5 and those in the SDI of 47.9. The ALSWH showed weight gain at mid-age independent of BMI to be associated with decreased physical health in the large population-based sample of Australian women in the ALSWH [26]. Over a two year period, women who were 45 to 50 years at baseline (47 to 52 years of age at follow-up), the same age range as the women in our intervention study, demonstrated that the women who lost the most weight over the two year period reported the best physical health (PCS = 50.9), which incrementally decreased for every category of weight gain up to those that gained 4.5 kg who had the poorest physical health (PCS = 48.7) [26]. Maintenance of SF-36 scores across the two years in the RCT participants is the positive in comparison with population trends for women at the same life stage.

There is only one menopausal weight gain prevention intervention that could be identified in the literature with which to compare the 40-Something results. The relatively labor-intensive WHLP intervention (15 sessions held over 20 weeks with regular follow-up over 4 years) also found a significant difference for weight after 54 months, with the intervention group losing 0.1 (5.2) kg and the control group gaining 2.4 (4.9) kg [8]. In that study, 55% of the intervention group (*n* = 260) were at or below baseline weight after the 54 months, compared with 26% of the control group. The WHLP study also found a significant intervention effect for waist circumference and body fat percentage, and their lifestyle intervention group also maintained lean body mass, which usually decreases with menopause [8]. The WHLP analyzed intervention effects according to baseline BMI and showed that there was only a significant difference between intervention and control group for the healthy weight women, which again echoes the 40-Something study results.

The intervention results should be considered in the context of the SCT framework on which they were based. As described in the methods, both the MI and SDI groups received components of behavioral capability in written materials, as well as the promotion of self-control through tools to monitor and regulate their own behaviors. Provision of these materials to both intervention groups may explain why both were successful at preventing obesity, regardless of intervention allocation. Other SCT components were delivered in the MI intervention only. The women in the MI group were provided with face-to-face support by the MI-trained health professionals using a client-centered counselling technique, to empower the participants to set their own goals and strategies, encourage change talk and reduce resistance to change. Activities and discussions were designed to build self-efficacy such as highlighting prior weight control successes of participants and helping to make goals achievable to allow the women to feel they were making progress. The use of a motivational interviewing style approach also provided an opportunity for participants to explore ambivalence to behavior change and to reflect on incongruencies between their personal values and beliefs and their current diet and physical activity behaviors, which may have further enhanced their intrinsic motivation to make and sustain behavior change.

The MI women also received special attention to maintaining their behavior change. In the final consultation session of the MI intervention, barriers to change, ways to overcome barriers, and strategies to stay motivated over the long-term were discussed. Our mediation analysis showed that compliance to the 10,000 steps per day recommendation mediated the effect on weight at 12 and 24 months, and compliance to the vegetables serve recommendation mediated the effect on weight at 24 months [28]. The authors hypothesize that the additional SCT and motivational interviewing components in the MI intervention enhanced self-efficacy and intrinsic motivation, and that this supported the MI women to maintain changes to their diet and physical activity behaviors, resulting in significantly lower weight, body fat and waist circumference measures in the MI group 12 months after all support was withdrawn. This is consistent with the evidence-based perspective that motivation is the best predictor of long-term success in weight control [29].

It is interesting that of the women who were overweight at baseline, more of those allocated to the MI group withdrew from the study before 24 months than those allocated to the SDI group. The opposite effect was observed with women who were of healthy weight at baseline, in that those allocated to the SDI group were more likely to withdraw than those allocated to the MI group. One possible conclusion is that the women who were a healthy weight at baseline responded to the motivational interviewing style of counselling more positively than some of the women who were overweight at baseline. All but one of the women overweight at baseline who remained in the study to 24 months were successful in achieving their weight goals, regardless of intervention allocation. The women who were overweight at baseline and allocated to the SDI group may have succeeded because they were already intrinsically motivated or they preferred to obtain information in writing rather than in a face-to-face consultation. Alternatively, the women who were overweight at baseline who withdrew from the study may have a habit of discontinuing a weight loss attempt after a short period from previous experience, which may have resulted in them giving up on seeking weight control independent of the intervention allocation.

It is important to acknowledge limitations of the current study. The main limitation is that the final numbers were relatively small because the rate of withdrawal from the intervention was higher than expected. This means that the study was likely to be under-powered for the main outcome measure. While there was no dropout bias by intervention group, and the monitoring phase was completed by approximately equal numbers of MI and SDI groups, there was a bias toward the healthy weight women being more likely to complete all stages of the intervention. Use of ITT analyses ameliorated this effect. Perhaps more concerning was the observation that self-reported inability to manage on income was also associated with women dropping out of the study before 12 months, although no women gave this as a reason for study withdrawal [14]. Since few intervention studies collect this measure, it is difficult to know how common this effect might be, but it does accord with observational studies that have found obesity and weight gain to be related to social class [30]. The same factors that make it difficult for women to prevent weight gain may make it difficult to participate in an RCT. No amount of intrinsic motivation can overcome economic disadvantage.

Despite these limitations, the study has implications for health service delivery in primary care. The MI intervention was intentionally based on the nationally funded model of health care delivery in Australia (Medicare), which allows people with a chronic and complex disease reimbursement for five allied health professional consultations annually [31]. The United States introduced a similar reimbursement scheme for the treatment of obesity in 2011 [32]. Unfortunately, women who are menopausal and overweight or at high risk of abdominal fat gain, would not typically be eligible to receive health service support through this model of care in either country. The 40-Something study findings illustrate the potential benefits of extending this model to application in the prevention of obesity, particularly abdominal obesity, and thus chronic disease at high risk life stages such as menopause, to prevent chronic disease rather than treating a person for chronic disease once it has developed. Other researchers have suggested that motivational interviewing be used in the primary care setting for weight loss [33], and a recent systematic review has shown dietitians to be more effective than other interventions within the primary care setting in achieving weight control [34]. Further intervention studies are needed with this relatively overlooked target group to examine the effectiveness, including cost-effectiveness, of weight gain prevention interventions to support healthy ageing.

## 5. Conclusions

The 40-Something RCT and monitoring study adds to the currently-limited evidence base related to weight control maintenance post intervention, especially during menopause transition. Weight control intervention effects need to be maintained to be effective and to do no harm to study participants and the broader community for whom they are intended. This relatively low intensity intervention, designed for women with a high risk of metabolically damaging weight gain, achieved a significant result for weight and waist circumference that was maintained for at least a further 12 months after intervention cessation. Even the minimal intensity intervention received by the control group prevented obesity, counter to the dominant population trend. A stronger focus on obesity prevention, rather than treatment, at this life stage has the potential to ameliorate the undesirable body composition changes associated with menopause, thereby decreasing the risk of metabolic syndrome and related chronic disease.

## Figures and Tables

**Table 1:** Baseline characteristics of participants according to level of completion.

Characteristics	Completed to 24 Months (*n* = 30)	Withdrew after 12 Months (*n* = 10)	Withdrew before 12 Months (*n* = 14)	Total *N* = 54	*p* Value
Allocation group					0.689
MI (*n*)	16	6	6	28	
SDI (*n*)	14	4	8	26	
Age (y) mean (Standard Deviation (SD))	47.3 (1.8)	47.3 (1.5)	47.1 (2.1)	47.3 (1.8)	0.905
Weight (kg) mean (SD)	66.8 (7.6)	69.0 (8.2)	72.2 (7.6)	68.7 (7.9)	0.103
Height (m) mean (SD)	1.65 (0.01)	1.65 (0.07)	1.67 (0.05)	1.65 (0.06)	0.575
Body fat % mean (SD)	34.8 (5.3)	35.6 (5.1)	38.4 (5.9)	35.9 (5.6)	0.123
Waist (cm) mean (SD)	81.7 (7.2)	83.9 (6.5)	86.0 (8.7)	83.1 (7.6)	0.190
Body Mass Index (kg/m^2^) mean (SD)	24.6 (2.3)	25.2 (1.9)	26.0 (2.8)	25.1 (2.4)	0.205
Body Mass Index 18.5–24.99 kg/m^2^ (*n*)	19 (MI = 12; SDI = 7)	5 (MI = 2; SDI = 3)	3 (MI = 0; SDI = 3)	27 (MI = 14; SDI = 13)	0.035
Body Mass Index 25–29.99 kg/m^2^ (*n*)	11 (MI = 4; SDI = 7)	5 (MI = 4; SDI = 1)	11 (MI=6; SDI=5)	27 (MI=14; SDI=13)	
Country of birth ^1^ *n* (%)					0.289
Australia	25 (83.3)	10 (100)	10 (71.4)	45 (84.9)	
Other	5 (16.7)	0 (0.0)	3 (21.4)	8 (15.1)	
Marital status ^1^ *n* (%)					0.494
Married/defacto,	23 (76.6)	9 (90.0)	10 (71.4)	41 (77.4)	
Divorced/separated/widowed/never married	7 (23.3)	1 (10.0)	3 (21.4)	12 (22.6)	
Highest qualification ^1^ *n* (%)					0.723
School only	6 (20.0)	2 (20.0)	4 (28.5)	12 (22.6)	
Post-school qualifications	24 (80.0)	8 (80.0)	9 (64.4)	41 (77.4)	
Work status ^1^ *n* (%)					0.123
Full/part time employment	28 (93.3)	10 (100)	10 (71.4)	48 (90.6)	
Home duties/care	2 (6.7)	0 (0.0)	1 (7.1)	3 (5.7)	
Full time student	0 (0.0)	0 (0.0)	2 (14.3)	2 (3.8)	
Difficulty managing on income ^1^ *n* (%)					0.004
Not too bad/easy	26 (86.7)	8 (80.0)	5 (35.7)	39 (73.6)	
Difficult sometimes/all times/impossible	4 (13.3)	2 (20.0)	8 (57.1)	14 (26.4)	
Self-rated health ^1^ *n* (%)					0.208
Excellent/very good/good	30 (100)	10 (100)	12 (85.7)	52 (98.1)	
Fair/poor	0 (0.0)	0 (0.0)	1 (7.1)	1 (1.9)	
Fruit and vegetable intake ^3^ mean (SD)					
Vegetable (serves)	2.78 (1.39)	2.84 (1.13)	2.56 (1.11)	2.74 (1.26)	0.857
Fruit (serves)	1.40 (0.81)	1.39 (0.80)	1.02 (0.72)	1.30 (0.79)	0.334
Physical Activity (IPAQ ^2^) *n* (%)					0.056
Low	3 (10.0)	1 (10.0)	5 (35.7)	10 (22.2)	
Moderate	8 (26.7)	4 (40.0)	5 (35.7)	16 (35.6)	
High	14 (46.7)	4 (40.0)	1 (7.1)	19 (42.2)	

^1^*n* = 27 (MI), *n* = 26 (SDI), *N* = 53 (Total) one MI respondent missing from those who withdrew before 12 months ^2^ *n* = 21 (MI), *n* = 24 (SDI), *N* = 45 (Total) nine participants answered ‘don’t know’ in response to at least one of the questions in the IPAQ survey at baseline and according to the scoring protocol their results were excluded from the analysis for this item. ^3^ Based on average daily intake from a 4-day weighed food record.

**Table 2:** Mean (SD) weight (kg) showing weight change according to intervention type and baseline BMI category (healthy weight and overweight) using intention to treat (ITT) analysis on 54 participants.

Data Collection Point	Group *N* = 54	MI *N* = 28	SDI *N* = 26	Unadjusted Difference *p* (95%CI)	Adjusted ^1^ Difference *p* Value
Baseline—group	68.7 (7.9)	68.7 (8.9)	68.6 (6.7)	0.982 (−4.30;4.39)	n/a
Healthy weight only	62.9 (5.3)	62.0 (6.4)	63.9 (3.7)	0.373 (−6.01;2.36)	n/a
Overweight only	74.4 (5.5)	75.3 (5.4)	73.4 (5.6)	0.365 (−2.41;6.32)	n/a
12 months—group	66.5 (7.7)	65.6 (8.5)	67.4 (6.7)	0.402 (−5.99;2.44)	0.034
Healthy weight only	61.6 (5.3)	59.5 (5.4)	63.8 (4.4)	0.032 (−8.23;−0.40)	0.002
Overweight only	71.5 (6.4)	71.8 (6.3)	71.1 (6.8)	0.764 (−4.42;5.95)	0.467
24 months—group	67.3 (8.0)	66.4 (8.7)	68.3 (7.2)	0.401 (−6.25;2.54)	0.024
Healthy weight only	62.0 (5.5)	60.1 (5.9)	64.0 (4.3)	0.063 (−8.05;0.23)	0.031
Overweight only	72.7 (6.4)	72.8 (6.0)	72.6 (7.1)	0.937 (−5.00;5.40)	0.197

^1^ Adjusted for weight at baseline. n/a: not applicable.

**Table 3:** Measures of metabolic syndrome according to intervention type (ITT analysis) (*n* = 54).

	Group *n* = 54 Mean (SD)	MI *n* = 28 Mean (SD)	SDI *n* = 26 Mean (SD)	Unadjusted Difference *p* (95% CI)	Adjusted ^1^ Difference *p* (95% CI)
SBP mmHg					
Baseline	120.3 (11.8)	120.7 (11.6)	119.9 (12.2)	0.807 (−5.755; 7.356)	n/a
12 months	119.1 (11.2)	117.6 (7.8)	120.7 (13.8)	0.316 (−9.249; 3.048)	0.137 (−8.341; 1.174)
24 months	113.1 (11.3)	111.1 (7.8)	115.1 (14.0)	0.201 (−10.204; 2.197)	0.114 (−9842; 1.094)
DBP mmHg					
Baseline	75.7 (8.3)	76.1 (7.0)	75.4 (9.6)	0.741 (−3.852; 5.381)	n/a
12 months	75.6 (7.3)	74.4 (5.5)	76.8 (8.7)	0.237 (−6.375; 1.613)	0.037 (−5.572; −0.175)
24 months	74.3 (7.4)	73.1 (6.1)	75.5 (8.4)	0.231 (−6.494; 1.634)	0.067 (−5.976; 0.206)
Body fat %					
Baseline	35.9 (5.6)	35.6 (5.8)	36.2 (5.4)	0.703 (−3.680; 2.500)	n/a
12 months	34.3 (5.5)	33.5 (6.0)	35.2 (4.9)	0.253 (−4.709; 1.264)	0.235 (−2.586; 0.650)
24 months	35.0 (5.8)	33.7 (6.5)	36.3 (4.8)	0.111 (−5.673; 0.5997)	0.030 (−3.455; −0.187)
Lean muscle %					
Baseline	27.4 (2.7)	27.5 (2.8)	27.2 (2.5)	0.649 (−1.154; 1.834)	n/a
12 months	27.8 (2.5)	28.1 (2.9)	27.6 (2.2)	0.432 (−0.844; 0.943)	0.592 (−0.607; 1.053)
24 months	27.6 (2.8)	28.1 (3.3)	27.1 (2.1)	0.231 (−6.494; 1.604)	0.119 (−0.182; 1.553)
Waist circumference cm					
Baseline	83.1 (7.6)	83.3 (8.2)	83.0 (7.0)	0.905 (−3.937; 4.440)	n/a
12 months	81.5 (8.1)	80.4 (8.6)	82.6 (7.5)	0.0307 (−6.705; 2.149)	0.045 (−4.941; −0.061)
24 months	80.6 (8.6)	79.0 (9.1)	82.3 (8.0)	0.163 (−8.015; 1.389)	0.006 (−6.046; −1.061)
VAT cm^3^					
12 months	79.5 (26.3)	75.6 (26.3)	84.4 (26.2)	0.296 (−25.737; 8.050)	n/a
24 months	84.5 (31.0)	79.3 (28.6)	90.9 (33.4)	0.241 (−31.485; 8.175)	0.601 (−10.894; 6.392)

^1^ Adjusted for baseline values except for visceral adipose tissue (VAT), which is adjusted for 12 month values and based on *n* = 40. n/a: not applicable.

**Table 4:** Short Form-36 (SF-36) summary scores across the three time points according to intervention type (ITT analysis) (*n* = 54).

		Group Mean (SD)	MI Mean (SD)	SDI Mean (SD)	Difference ^1^
PCS	Baseline	50.6 (6.9)	51.6 (5.5)	49.4 (8.1)	0.451
	12 months	50.7 (7.2)	51.8 (7.2)	49.8 (7.8)	0.374
	24 months	50.2 (7.2)	51.5 (6.1)	47.9 (7.3)	0.107
MCS	Baseline	49.4 (9.2)	47.8 (10.7)	51.2 (7.0)	0.502
	12 months	51.4 (10.1)	49.9 (11.0)	52.0 (9.7)	0.762
	24 months	49.7 (11.3)	48.7 (12.0)	51.5 (9.1)	0.840

^1^ Independent samples, Mann Whitney *U*. PCS = Physical Component Summary Score; MCS = Mental Component Summary Score.
